# A literature review on the potential clinical implications of streptococci in gastric cancer

**DOI:** 10.3389/fmicb.2022.1010465

**Published:** 2022-10-26

**Authors:** Mengli Zi, Yanqiang Zhang, Can Hu, Shengjie Zhang, Jinxia Chen, Li Yuan, Xiangdong Cheng

**Affiliations:** ^1^Department of Gastric surgery, The Cancer Hospital of the University of Chinese Academy of Sciences (Zhejiang Cancer Hospital), Institutes of Basic Medicine and Cancer (IBMC), Chinese Academy of Sciences, Hangzhou, China; ^2^Zhejiang Provincial Research Center for Upper Gastrointestinal Tract Cancer, Zhejiang Cancer Hospital, Hangzhou, China; ^3^Zhejiang Key Lab of Prevention, Diagnosis and Therapy of Upper Gastrointestinal Cancer, Zhejiang Cancer Hospital, Hangzhou, China

**Keywords:** gastric cancer, Streptococcus, Helicobacter pylori, digestive tract, oral

## Abstract

Streptococcus is widely found in nature and the human body, and most species are not pathogenic. In recent years, studies have found that Streptococcus is associated with gastric cancer. Streptococcus was found to be enriched in the oral cavity, stomach and intestine of gastric cancer patients and found to be increased in gastric cancer tissues, suggesting that Streptococcus may be the pathogenic bacteria underlying gastric cancer. This review discusses the discovery of Streptococcus, the relationship between Streptococcus and gastric cancer, and the possible carcinogenic mechanism of Streptococcus and summarizes the progress of the research on the role of Streptococcus in gastric cancer to provide new ideas for the early detection, diagnosis and treatment of gastric cancer.

## Introduction

Gastric cancer is the fifth most common type of malignancy in the world and the fourth leading cause of death from cancer (Sung et al., 2021). Asia (and mainly China) exhibits the highest number of gastric cancer cases (Torre et al., 2016; Zhao et al., 2017), and the 5-year survival rate of gastric cancer patients is 27.4% in China (Huang et al., 2021), making it one of the major cancers threatening human health. Gastric cancer is a multifactorial and multistep inflammatory disease. It is believed that the development process of gastric cancer is as follows: chronic superficial gastritis, chronic atrophic gastritis, intestinal metaplasia, atypical hyperplasia, gastric adenocarcinoma (Correa, 1992). Studies have found that multiple factors, including host genetic factors (El-Omar et al., 2000; Allison and Ferrero, 2010; Castaño-Rodríguez et al., 2014; Mommersteeg et al., 2018), environmental factors (such as smoking, alcohol consumption, high salt and meat intake, low vegetable/fruit intake) and microbial factors (such as Helicobacter pylori infection and other gastric microorganisms), play an important role in gastric cancer (Correa, 1995; Correa and Houghton, 2007; Moss, 2017). Gastric cancer is mainly treated by surgery but also by chemotherapy, immunization and targeted drug therapy (Ajani et al., 2022), while anti-Helicobacter pylori infection is currently the only microbial treatment for gastric cancer prevention (Wu et al., 2010).

Some epidemiological studies have shown that gastric microbes are involved in the occurrence of gastric cancer by inducing chronic inflammation or downregulating host immunity (Parsonnet, 1995). For example, Helicobacter pylori (Hp), classified as a class I carcinogen by the World Health Organization, destroys the structure and function of the gastric epithelium by enhancing the inflammatory response (Amieva and Peek, 2016), affects the richness and evenness of other bacterial communities (Bessède and Mégraud, 2022)and plays a key role in the initial steps of gastric cancer. However, studies have found that gastric cancer still occurs after the eradication of H. pylori (Fukase et al., 2008; Ma et al., 2012), less than 3% of people infected with H. pylori develop gastric cancer (Engstrand and Graham, 2020), and the level of H. pylori colonization decreases and its presence eventually disappears with the progression of gastric cancer (El-Omar et al., 1997). At the same time, studies using the INS-GAS mouse model showed that stomach and intestinal microbes could promote the formation of tumor lesions (Bik et al., 2006; Lofgren et al., 2011; Maldonado-Contreras et al., 2011; Lertpiriyapong et al., 2014). These results indicate that bacteria other than H. pylori also play an important role in the occurrence and development of gastric cancer. With the development of molecular biology and metagenomics, scientists have a more comprehensive understanding of gastrointestinal microbes, and it is believed that microbial dysbiosis can promote the occurrence of gastric cancer through different mechanisms, and Streptococcus bacteria are considered to affect the development of cancers in the oral cavity, lungs, colorectum and cervix (Kang et al., 2021; Stasiewicz and Karpiński, 2021; Goto, 2022; Karpiński et al., 2022).

Studies have found that the flora of patients with gastric cancer is in an imbalanced state, and Streptococcus is enriched in gastric cancer tissues (Liu et al., 2019; Shao et al., 2019; Dai et al., 2021), which is significantly different from the flora of healthy people or patients with chronic gastritis (Eun et al., 2014; Coker et al., 2018). Therefore, Streptococcus is considered a potential marker for predicting gastric cancer (Qi et al., 2019). Yu et al. used a random forest model (RF) to produce further evidence of the use of Streptococcus as a marker of gastric cancer (Yu et al., 2021). Both H. pylori and Streptococcus can produce urease, which is the main inducer of the innate immune response and is involved in the occurrence of gastric cancer (Mobley and Hausinger, 1989; MacMicking et al., 1997; Gobert et al., 2002; Suerbaum and Michetti, 2002; Brandi et al., 2006; Osaki et al., 2008); Streptococcus is also involved in the formation of nitroso compounds (NOCs) in the stomach (Ayanaba and Alexander, 1973; Jo et al., 2016; Sohn et al., 2017), and NOCs are associated with an increased risk of gastric cancer (Ayanaba and Alexander, 1973; Mowat et al., 2000; Dicksved et al., 2009; Jo et al., 2016). These results indicate that Streptococcus may affect the occurrence and development of gastric cancer. Moreover, studies on gastric cancer-related microorganisms are not limited to the stomach but have also been conducted on the oral cavity and intestine, and Streptococcus has been found in different studies of the three sites, suggesting the important role of Streptococcus in gastric cancer research.

## Streptococcus in gastric microecology

Streptococcus is another common bacterial pyogenic coccus that widely exists in nature. The important Streptococcus encountered in medicine mainly include alpha-hemolytic streptococci, beta-hemolytic streptococci, and non-hemolytic streptococci. Streptococcus belongs to the bacterial domain, Firmicutes phylum, Bacillus class, Lactobacillus order, Streptococcus family, and Streptococcus genus and is further subdivided into different species of Streptococcus. Streptococcus is a microorganism that naturally exists in the human body, especially in the digestive tract. The Streptococcus genus and its different species, such as Streptococcus pneumoniae, Streptococcus pyogenes and Streptococcus agalactiae, have been found in healthy people and patients with gastric cancer (Li et al., 2009; Delgado et al., 2013; Sohn et al., 2017; Coker et al., 2018; Chen et al., 2019).

Due to its highly acidic environment, motility and mucosal mucus layer, the stomach was regarded as a sterile environment until the discovery of Helicobacter pylori (Hp) in 1982, after which Hp was considered the only bacterium that could colonize the stomach. However, in 1981, a few months before the discovery of H. pylori, the Lancet reported that a large number of bacteria, including Streptococcus, Neisseria and Lactobacillus, could be detected in the stomach, and multiple studies have found streptococci in gastric juice. In 1984, Sharma et al. performed bacterial culture using the gastric juice of healthy men and found 9 bacterial genera, including Streptococcus (hemolytic and nonhemolytic) (Sharma et al., 1984); this was the first time Streptococcus was cultured using gastric juice. Sjostedt et al. cultured Streptococcus using the gastric juice of gastric cancer patients in the following year (Sjöstedt et al., 1985). Later, Choi and Hu et al. performed metagenomic analysis of gastric juice and found the presence of Streptococcus, which was significantly increased in gastric cancer patients (Choi et al., 2017; Hu et al., 2018). Multiple studies have found streptococcal overgrowth in gastric juices during proton pump inhibitor (PPI) acid-suppressive therapy (Thorens et al., 1996; Sanduleanu et al., 2001; Rosen et al., 2014; Rosen et al., 2015; Tsuda et al., 2015).

**Figure 1:**
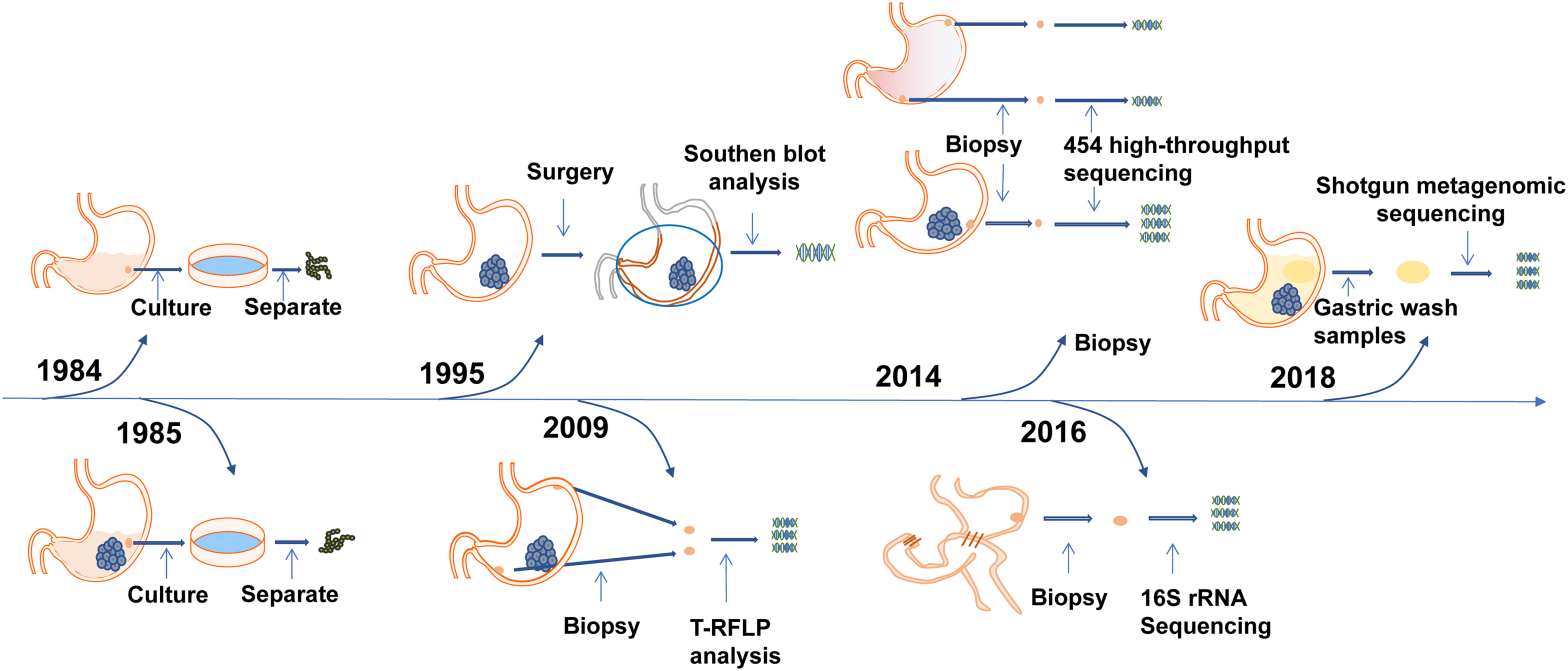
Time axis diagram: Events of great significance in the study of Streptococcus and gastric cancer. Pound’s team was the first to grow the Streptococcus genus in the stomach juices of healthy men in 1984. Nord’s team grew Streptococcus in gastric juice from patients with stomach cancer in 1985. Terraada’s team found DNA fragments of Streptococcus anginosus in gastric cancer surgical specimens in 1995. Engstrand’s team first used molecular biology techniques to analyze the microbiota of gastric cancer and found that Streptococcus was the dominant bacterium in 2009. Kim’s team first found that gastric microbes were different from those of patients with chronic gastritis, and the Streptococcus family was increased in gastric cancer in 2014. Wu’s team first studied the gastric microbiota after gastric cancer surgery and found that Streptococcus was still one of the most abundant bacterial genera in 2016. Metagenomic analysis of gastric fluid by Zhang’s team found that Streptococcus was the dominant genus and that its abundance was significantly increased in patients with gastric cancer in 2018.

To further confirm the relationship between Streptococcus and gastric cancer, bacterial detection and analysis of gastric mucosa tissues have also been carried out. Sasaki et al. performed Southern blot analysis on surgical specimens of gastric cancers in 1995 and detected DNA fragments of Streptococcus anginosus in 9 (20%) surgical specimens (Sasaki et al., 1995). Three years later, they conducted research in the same way and found the presence of Streptococcus anginosus in the cancerous gastric tissues but not in the adjacent normal tissues (Sasaki et al., 1998). The results of a study by Dicksved et al. showed that the flora observed in gastric cancer mainly comprised different species of Streptococcus, Lactobacillus, Veillonella and Prevotella (Dicksved et al., 2009). Eun, Jo, and Coker et al. found that the abundance of Streptococcus was significantly increased in gastric cancer patients (Ayanaba and Alexander, 1973; Eun et al., 2014; Coker et al., 2018). The first study of gastric microbiota after subtotal gastrectomy in patients with gastric cancer by Tseng et al. found that Streptococcus remains one of the most abundant bacterial genera (Tseng et al., 2016). The important events in the discovery of Streptococcus in the gastric microecological environment are shown in Figure 1. Streptococcus was found in the gastric juice and gastric mucosa of healthy people and patients with gastric cancer and was enriched in gastric cancer patients, while Streptococcus may be present in the oropharynx and enter the stomach through food swallowing and was found to be a transit bacterium. To further investigate whether Streptococcus colonizes the stomach, in 2009, Li et al. obtained biopsy samples extracted from gastritis patients and healthy controls that were washed in phosphate buffered saline (PBS). After three consecutive washes, more than 90% of bacteria, including Streptococcus, were still attached to the specimens (Li et al., 2009). A high bacterial isolation rate (average 56.5%) observed in a 2014 study suggested that Streptococcus may colonize the stomach, not just pass through it (Khosravi et al., 2014). In 2020, Spiegelhauer et al. used 16S rRNA sequencing for the first time aiming to distinguish between transient and resident bacteria, and the results suggested that Streptococcus may be a resident bacteria (Spiegelhauer et al., 2020). The above studies show that Streptococcus exists in the gastric mucosa and is a persistent bacterium. It is enriched in gastric cancer and may be related to the occurrence and development of gastric cancer, which is worthy of further research.

## Relationship between Streptococcus and Helicobacter pylori

The gastric microbiota of patients infected with Helicobacter pylori (Hp) is different from that of noninfected patients, and studies have shown that Hp is the main factor that influences the dysbiosis of the gastric microbiota (Wang L. et al., 2016; Liu et al., 2019; Guo et al., 2020). The relationship between Streptococcus, one of the dominant bacteria in the stomach, and Hp is unclear. Researchers have studied the relationship between intragastric Streptococcus and Hp in nongastric cancer patients in terms of the presence/absence of Hp and bactericidal or acid-suppressive therapy. First, bacteria other than Hp in the gastric mucosa of nongastric cancer patients were analyzed under the premise of the existence of Hp, and it was found that the dominant bacterial species were Neisseria flavescens (13.7%), Streptococcus salivarius (9.5%), Rothia mucilaginosa (8.9%) and Streptococcus pneumonia (6.6%) (Hu et al., 2012). Moreover, the abundances of Streptococcus, Prevotella and Haemophilus in the stomach increased significantly during Hp sterilization treatment or PPI alone, but the bacterial species that were present did not change significantly, and the relative proportion of existing bacteria changed and recovered to the pretreatment level for a period of time after treatment (Stark et al., 1996; Thorens et al., 1996; Adamsson et al., 1999; Rosen et al., 2014; Rosen et al., 2015). A study in nongastric cancer patients without Hp infection found that Streptococcus and Prevotella were relatively abundant (Li et al., 2009). Analysis of the cooccurrence network of gastric microorganisms in chronic gastritis patients showed that there was a significant negative correlation between the abundances of H. pylori and Streptococcus (Parsons et al., 2017). The above studies showed that Streptococcus did not overgrow in the presence of Hp, while gastric acid secretion was inhibited or Streptococcus abundance increased during bactericidal treatment; these findings indicate that Streptococcus was affected by Hp and gastric acid secretion. These studies showed that Streptococcus did not grow in the presence of Hp, while the increase in Streptococcus abundance during the inhibition of gastric acid secretion or bactericidal treatment indicated that Streptococcus abundance was affected by Hp and gastric acid secretion.

The above studies have shown that Streptococcus and Hp are closely correlated in nongastric cancer patients, and some studies have also shown that Streptococcus and Hp are closely correlated in gastric cancer patients. A study in 2016 found that Hp was the most dominant bacterium and that Streptococcus was the second most dominant bacterium in Hp-positive gastric cancer patients (Jo et al., 2016). In the following year, Sohn et al. conducted a study on Hp-negative gastric cancer. According to the overlap analysis of non-Hp urease-producing bacteria and non-Hp nitrate-reducing bacteria, Streptococcus accounted for the largest proportion in Hp-negative gastric cancer at the family level, while Streptococcus pseudopneumoniae, Streptococcus parasanguinis, and Streptococcus oralis accounted for a larger proportion at the species level (Sohn et al., 2017). In the absence of Hp infection, Streptococcus is prominent in gastric cancer and can be considered the pathogenic bacteria underlying gastric cancer. Another study also suggested that Streptococcus and Neisseria may play a role in the development of gastric cancer (Gantuya et al., 2019). Most gastric cancers are Hp-positive gastric cancers, so studies of Streptococcus are affected by Hp. Although Hp was excluded from the analysis of the data, the authenticity and validity of the data were also affected. Although the number of Hp-negative gastric cancer samples was small and few studies were conducted, the influence of Hp could be excluded, which is of great significance for Streptococcus research. Since Hp is a recognized pathogen underlying gastric cancer, it is further speculated that Streptococcus may work together with Hp or play a role in different stages of gastric cancer.

## Changes in Streptococcus in the digestive tract during the occurrence and development of gastric cancer

**Figure 2:**
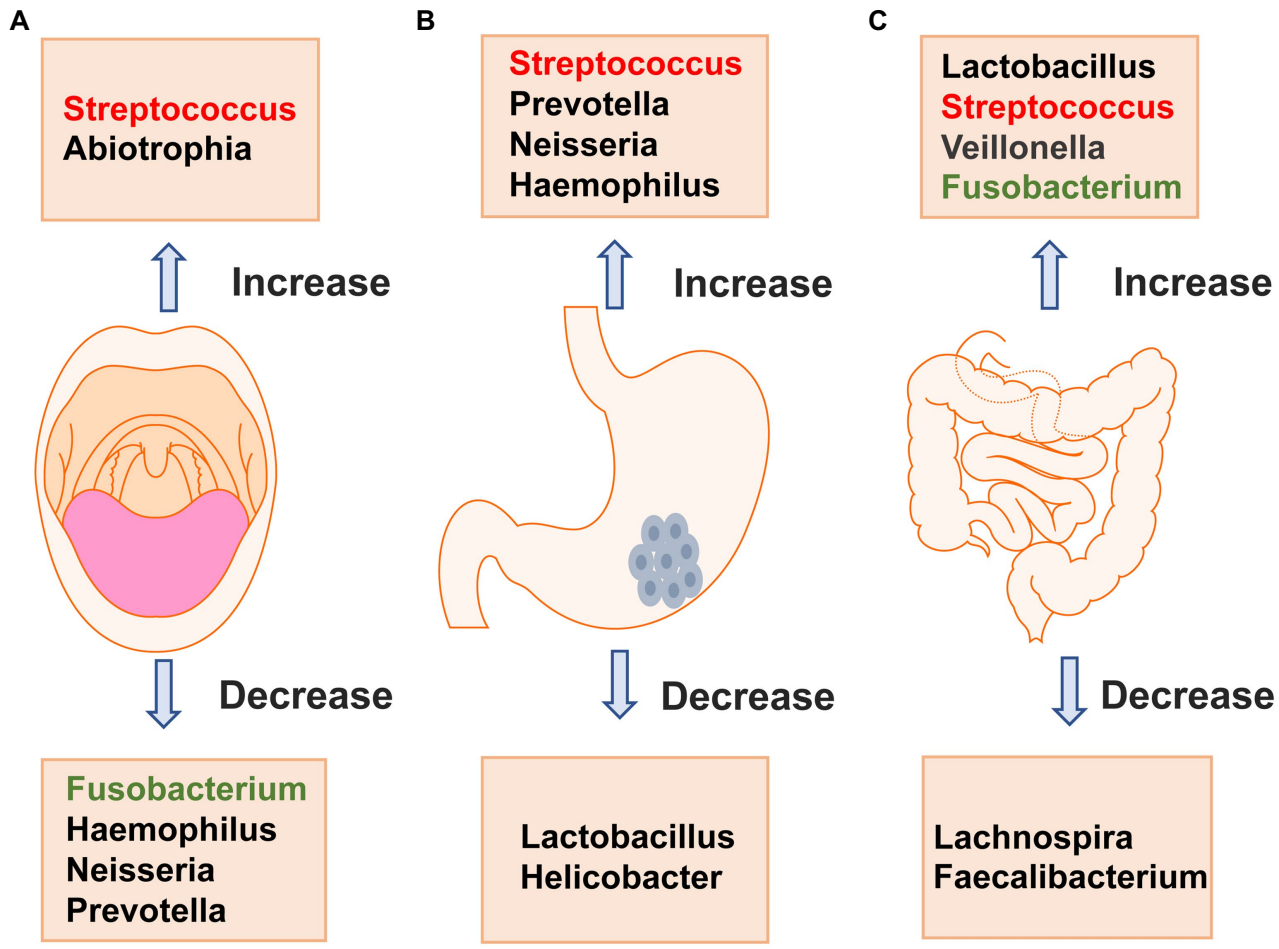
Changes in major bacteria in the oral cavity **(A)**, stomach **(B)** and intestinal tract **(C)** in patients with gastric cancer. **A** shows that the changes in oral microbiota of gastric cancer patients, Streptococcus and Abiotrophia are significantly increased compared with healthy people, while Fusobacterium, Haemophilus, Neisseria, and Prevotella are significantly decreased. **B** shows that the changes in major bacteria in stomach of patients with gastric cancer, Streptococcus,Prevotella, Neisseria,and Haemophilus are significantly increased compared with healthy people, while Lactobacillus and Helicobacter are significantly decreased. **C** shows that the changes in major bacteria in intestinal tract of patients with gastric cancer, Lactobacillus, Streptococcus, Veillonella and Fusobacterium are significantly increased compared with healthy people, while Lachnospira and Faecalibacterium are significantly decreased.

The digestive tract consists of the mouth, pharynx, esophagus, stomach and intestines, and streptococci exist in various parts of the digestive tract. Streptococcus in different parts of the digestive tract has been studied in gastric cancer. Next, we discuss the changes in Streptococcus in the occurrence and development of gastric cancer from the perspective of the oral cavity, stomach and intestinal tract. Studies have shown that Streptococcus exists in the oral cavity of healthy people and is obviously enriched in gastric cancer, but different species of Streptococcus exhibit different changes during gastric cancer. Streptococcus in the stomach also accumulates gradually during the progression from chronic gastritis to atrophic gastritis and finally to gastric cancer and is expected to become a marker for the diagnosis of gastric cancer. The intestinal flora is complex and diverse, and Streptococcus abundance is significantly increased in the intestinal tract of patients with gastric cancer. The difference in Streptococcus in the feces of patients with chronic gastritis and gastric cancer can be used to distinguish them, providing a supplement for noninvasive examination methods for early diagnosis. The changes that occur in the main bacteria of the oral cavity, stomach and intestinal tract of patients with gastric cancer are shown in Figure 2. We will review the changes that occur in Streptococcus in the oral cavity, stomach and intestinal tract during the occurrence and development of gastric cancer.

### Changes in oral Streptococcus during the occurrence and development of gastric cancer

The oral cavity is the starting point of the human digestive tract and is home to a variety of bacterial communities, including at least 11 phyla and 70 genera (Ahn et al., 2012). The oral microbiome may affect bacteria in the esophagus, stomach and gut; for example, some lactobacilli found in human feces are heterologous to the gut, originating from the oral cavity (Dal Bello and Hertel, 2006). A large number of bacteria in the stomach are also the dominant bacteria in the oral cavity, which may be microorganisms that are present during swallowing (Andersson et al., 2008). The oral microbiome is the second most complex microbial community in the human body and plays an important role in oral and systemic health. For example, Ndegwa et al. performed a prospective study and found that poor oral health was associated with an increased risk of gastric cancer (Ndegwa et al., 2018).

Studies in healthy volunteers show that streptococci are the dominant bacteria in the oral flora. Mowat and Zilberstein et al. conducted bacterial culture on oral specimens and found that the most common bacteria was α-hemolytic Streptococcus, which appeared most frequently in saliva (Mowat et al., 2000; Zilberstein et al., 2007). Andersson et al. performed 454 pyrosequencing on the highly variable region of 16S rRNA in throat specimens and found that Streptococcus was the dominant genus, followed by Prevotella (Andersson et al., 2008). Tsuda et al. analyzed the saliva of subjects taking PPIs using bacterial culture and high-throughput sequencing methods and found that Streptococcus was the most abundant and that PPI intake did not affect the results (Tsuda et al., 2015). All the above findings suggest that Streptococcus is dominant in the oral flora and is not affected by PPIs. Since quantities of Streptococcus exist in the oral cavity, they may enter the stomach through the swallowing of food, affecting the flora in the stomach and leading to the occurrence of gastric cancer.

Streptococcus is present in the oral cavity of healthy people, so we wondered whether the Streptococcus in the oral cavity of patients with precancerous lesions and gastric cancer were different. The researchers studied the oral flora of patients with precancerous gastric cancer lesions and detected DNA fragments of Streptococcus mutans in saliva (Salazar et al., 2013). Patients with gastric intestinal metaplasia exhibited an enrichment in the oral species Peptostreptococcus stomatis, whereas Streptococcus mutans, S parasanguinis and S sanguinis abundances were reduced (Wu et al., 2021). These findings suggest that a certain Streptococcus species may play a role in the development of gastric precancerous lesions. At the species level, Streptococcus shows different changes in precancerous lesions. Whether Streptococcus changes again when it develops to gastric cancer is unknown. Wu et al. conducted 16S rRNA sequencing on tongue coating samples from 57 gastric cancer patients in 2018, and the results showed that the relative abundance of Streptococcus was relatively high in gastric cancer patients, indicating that Streptococcus is a common risk factor and has a potential carcinogenic effect (Wu et al., 2018), but the causal relationship between Streptococcus and the occurrence of gastric cancer could not be verified. The following year, Japanese scholars performed 16S rRNA sequencing on saliva samples obtained from 59 patients with digestive tract cancer and 118 controls and found that the relative abundances of Streptococcus sanguinis and Streptococcus parasanguinis in gastric cancer patients were relatively low (Kageyama et al., 2019). Comparing Streptococcus changes during gastric carcinogenesis, Huang et al. used 16S rRNA sequencing to obtain a salivary microbiome map and established a random forest model to classify gastric tissue types in 2021. They found that the abundance of Peptostreptococcus in saliva gradually decreased during the progression from superficial gastritis to atrophic gastritis to gastric cancer, but the abundance of the Streptococcus genus was significantly increased in gastric cancer and was the most representative bacterial genus (Huang et al., 2021). It was proposed for the first time that Streptococcus may be an indicator for the diagnosis of gastric cancer. Studies of different species of Streptococcus have shown that intraoral Streptococcus changes at different stages of gastric disease and may be a predictor of gastric cancer (Table 1). However, whether the changes in Streptococcus are the cause or the result of gastric cancer is still inconclusive, and the pathogenic mechanism is not clear.

**Table 1:** Changes in Streptococcus in the oral cavity.

Author	Year	Subjects	Region/country	Method	Samples	Main findings	Reference
Wu	2018	57 patients with gastric adenocarcinoma and 80 healthy controls	Jiangsu Province, China	16S rRNA gene sequencing	The tongue coating samples	The relative abundance of streptococcus was higher in gastric cancer patients. The relative abundance of streptococcus was also higher in noncardiac cancer patients than in the controls, however, the effect of streptococcus risk was not significant in cardiac cancer patients.	Wu et al., 2018
Kageyama	2019	59 patients with cancer in any part of the digestive tract (tongue/pharynx, esophagus, stomach, and large intestine), and 118 matched controls	Japan	16S rRNA gene sequencing	Saliva	The relative abundances of Streptococcus sanguinis and Streptococcus parasanguinis in gastric cancer patients were low.	Kageyama et al., 2019
Huang	2021	superficial gastritis (SG), atrophic gastritis (AG), gastric cancer (GC)	Beijing, China	16S rRNA gene sequencing	Saliva	At the genus level, Prevotella, Haemophilus and Streptococcus constitute more than 70% of the salivary flora at each histological stage of GC; Peptostreptococcus abundance gradually decreased from SG → AG → GC; The abundance of the Streptococcus genus was significantly increased in GC while Peptostreptococcus was enriched in SG.	Huang et al., 2021
Wu	2021	89 patients with intestinal metaplasia and 89 healthy controls	New York	Metagenomics Sequencing	Oral wash samples	The abundance of oral species Peptostreptococcus stomatis was higher in the case group than in the control group, while Streptococcus mutans, S.parasanguinis and S.sanguinis abundances were lower.	Wu et al., 2021

### Changes in endogastric Streptococcus during the occurrence and development of gastric cancer

Due to gastric motility, the presence of the mucus layer, low pH, and acid secretion, it was initially thought that no bacteria could survive the hostile gastric environment (Bik et al., 2006; O’Hara and Shanahan, 2006; Nardone and Compare, 2015). After the discovery of Hp, a large number of studies have shown that other bacteria also exist in the stomach and can colonize the stomach instead of just passing through (Li et al., 2009; Khosravi et al., 2014; Spiegelhauer et al., 2020). With the advancement of molecular biotechnology and gene sequencing technology, the mystery of the gastric microbiota has been gradually revealed. The existence of gastric microbiota was also confirmed to promote the occurrence of gastric cancer in an INS-GAS mouse model (Lofgren et al., 2011; Lertpiriyapong et al., 2014; Shen et al., 2022). The study of the relationship between gastric microorganisms and gastric cancer has also become a hot topic in recent years, with much progress in the diagnosis and microbial treatment of early gastric cancer being expected to occur.

Studies have shown that Streptococcus exists in the stomach of healthy people (Sharma et al., 1984; Monstein et al., 2000; Mowat et al., 2000; Kato et al., 2006; Zilberstein et al., 2007) and is the dominant bacterium other than Hp (Seo et al., 2014); in addition, it was also found that Streptococcus abundance was significantly increased in gastric cancer, and Streptococcus remained one of the most abundant genera after subtotal gastrectomy (Tseng et al., 2016). Since the first discovery of DNA fragments of Streptococcus anginosusin gastric cancer tissues in 1998 (Sasaki et al., 1998), subsequent studies have used 16S rRNA sequencing to identify microorganisms to ensure that the analyzed bacteria are live bacteria, which can partially mitigate the impact of upstream oral microorganisms on research results. In 2009, Dicksved et al. found that the gastric cancer flora mainly comprised different species of Streptococcus, Lactobacillus, Veillonella and Prevotella (Dicksved et al., 2009). In 2016, Jo et al. divided the research subjects into four groups: Hp (+) gastric cancer, Hp (−) gastric cancer, Hp (+) nongastric cancer and Hp (−) nongastric cancer. The study found that the abundance of Streptococcus ranked second in all four groups (Jo et al., 2016). Metagenomic analysis of bacteria and extracellular vesicles conducted by Choi et al. the following year found that Helicobacter pylori and Streptococcus were two major bacterial genera, and their abundances increased significantly in gastric cancer patients (Choi et al., 2017). In 2020, Gunathilake et al. conducted a study at the Streptococcus species level and found that Streptococcus CP003667 and Streptococcus vestibularis were enriched in the healthy control group, while Streptococcus NCVM was enriched in the gastric cancer group (Gunathilake et al., 2020). In 2021, Pimentel-nunes et al. found that the abundance of Dicoccus, especially Streptococcus, was significantly increased in patients with early-stage gastric cancer (Pimentel-Nunes et al., 2021). In the same year, Gunathilake et al. also conducted a study on early gastric cancer and found that the abundances of Streptococcus vestibularis and Peptostreptococcus stomatis decreased significantly in the gastric cancer group (Gunathilake et al., 2021). The above studies showed that the abundance of Streptococcus was significantly increased in gastric cancer at the genus level, but at the species level, the abundances of some Streptococcus species were decreased in gastric cancer.

All the above studies have regarded the whole stomach as a microecological environment, while some researchers believe that different parts of the stomach are different microecological environments, so it is necessary to compare whether the Streptococcus in cancerous gastric tissue and adjacent tissue are different. In 2019, Chen et al. first described the microbial cooccurrence network in the cancerous tissues and adjacent tissues of gastric cancer patients and found that the enriched bacterial groups in cancer tissues were dominated by oral bacteria (such as Peptostreptococcus, Streptococcus, and Fusobacterium), while in adjacent noncancer tissues, lactic acid-producing bacteria (such as Lactococcus lactis and Lactobacillus brevis) were more abundant (Chen et al., 2019). In the same year, Liu et al. found that Helicobacter pylori abundance was significantly reduced in cancer tissue, while Streptococcus anginosus abundance was significantly increased (Liu et al., 2019). Later, Dai et al. found that the abundance of Streptococcus in cancerous tissues increased (Dai et al., 2021). Shao et al. studied the cancerous tissue and adjacent noncancerous tissue of cardia adenocarcinoma patients and found that at the genus level, the relative abundance of Streptococcus in the cancerous tissue was high, and the relative abundance of Helicobacter pylori was low (Shao et al., 2019). The above results indicated that the location of gastric cancer and the microecological environment did not affect the enrichment of Streptococcus.

It is believed that progression from chronic nonatrophic gastritis to chronic atrophic gastritis to intestinal metaplasia to dysplasia to gastric cancer is a common process in the occurrence and development of gastric cancer. Therefore, many researchers began to study the changes in the microbiota from the precancerous lesions of gastric cancer, in the context of achieving early prevention, early diagnosis and early treatment of gastric cancer using an analysis of microorganisms before the occurrence of gastric cancer. A 2018 study found that Streptococcus was most abundant in the microbiota of patients with chronic gastritis (Ferreira et al., 2018). Conti et al. found that Streptococcus was more common in gastritis patients with atrophic gastritis, and Streptococcus was positively correlated with OLGA/OLGIM stages of chronic gastritis (Conti et al., 2021). A 2021 study in New York conducted by Wu et al. found that the abundances of Streptococcus mutans, Streptococcus parahaemolyticus, and Streptococcus sanguinis were lower in the gastric mucosa of patients with intestinal metaplasia than healthy individuals (Wu et al., 2021). In the same year, a study conducted in and around Anhui, China, found that Streptococcus had a high centrality in the progression of gastric precancerous lesions (Liu D. et al., 2021). In 2018, Coker et al. conducted a microbial study on patients with superficial gastritis (SG), atrophic gastritis (AG), intestinal metaplasia (IM) and gastric cancer (GC) in Xi’an, China, and validated the results in Inner Mongolia. The study found that Peptostreptococcus stomatis and Streptococcus anginosus have significant centrality in the gastric cancer ecological network, the area under the curve (AUC) value for distinguishing gastric cancer from superficial gastritis was 0.82, and the AUC obtained in the validation cohort was 0.81 (Coker et al., 2018). In 2021, Pimentel-Nunes et al. conducted a microbial analysis of healthy controls, patients with advanced atrophic gastritis with intestinal metaplasia, and early-stage gastric cancer. The study found that from controls to patients with intestinal metaplasia and then to patients with gastric cancer, Streptococcus abundance increased gradually from 19.3 to 33.7%, and Streptococcus is the predominant bacteria in early-stage gastric cancer (Pimentel-Nunes et al., 2021). The study of intragastric Streptococcus in gastric cancer is shown in Table 2. Streptococcus was enriched during disease progression, and this change was more pronounced and statistically significant when gastric cancer patients were compared with chronic gastritis patients to distinguish the two conditions. Although it is not clear whether the changes in Streptococcus are the cause or effect of gastric cancer, it is significant for the diagnosis of early-stage gastric cancer.

**Table 2:** The study of intragastric Streptococcus in gastric cancer.

Author	Year	Subjects	Region/country	Method	Samples	Main findings	Reference
Sjöstedt	1985	Patients with gastric ulcer, duodenal ulcer, gastritis, gastric cancer, postoperative gastric cancer patients and healthy controls, 10 per condition	Sweden	Bacterial culture	Saliva, esophageal fluid, gastric fluid	Streptococcus was isolated from gastric juice cultures of patients with gastritis and gastric cancer and from those who underwent gastrectomy.	Sjöstedt et al., 1985
Sasaki	1995	43 patients with gastric cancer	Japan	Southern blot analysis and 16S rDNA sequencing	Surgical specimens	DNA fragments of Streptococcus anginosus were found in 9 (20%) surgical specimens	Sasaki et al., 1995
Sasaki	1998	15 esophageal cancer, 43 gastric cancer, 16 lung cancer, 10 cervical cancer, 14 renal cell cancer, 10 colorectal cancer, 19 bladder cancer patients	Japan	Southern blot analysis, the 16S rDNA of streptococcus anginosus was analyzed by PCR	Cancer tissue and adjacent noncancer tissue	DNA fragments of Streptococcus anginosus were found in DNA samples of cancer tissues of esophagus and gastric cancers, but not in adjacent noncancer tissue.	Sasaki et al., 1998
Dicksved	2009	10 patients with gastric cancer, 5 dyspeptic control patients	Sweden	16S rRNA sequencing	Stomach biopsies	The gastric cancer microbiota was instead dominated by different species of the genera Streptococcus, Lactobacillus, Veillonella and Prevotella	Dicksved et al., 2009
Aviles-Jimenez	2014	5 patients with chronic nonatrophic gastritis, 5 patients with intestinal metaplasia, and 5 patients with gastric cancer	Mexico	G3 chip was used to extract DNA for microflora analysis	Stomach biopsies, surgical specimens	Lachnospiraceae and Streptococcaceae representing over 20% of families in patients from all three disease groups.	Aviles-Jimenez et al., 2014
Jo	2016	HP-negative control group (*n* = 13), HP-positive control group (*n* = 16), HP negative cancer group (*n* = 19), and HP-positive cancer group (*n* = 15)	Korea	Barcoded 454 Pyrosequencing of the 16S rRNA Gene	Gastric mucosal (antrum and corpus) biopsies	Streptococcus ranked second in all four groups; in the high intestinal metaplasia group, the proportion of streptococcus increased.	Jo et al., 2016
Sohn	2017	HP-negative control group (*n* = 2), HP-positive control group (*n* = 3), HP negative cancer group (*n* = 2), and HP-positive cancer group (*n* = 5)	Korea	Bar-coded 454 pyrosequencing of the 16S rRNA gene	Antrum and body biopsy	The higher composition of Streptococcus pseudopneumoniae, S. parasanguinis, and S. oralis in Hp (−) cancer groups than the others, only in the body. At the family level, streptococcus accounted for the largest proportion of Hp-negative gastric cancers.	Sohn et al., 2017
Coker	2018	81 patients with superficial gastritis (SG), atrophic gastritis (AG), intestinal metaplasia (IM) and gastric cancer (GC), 126 cases from inner Mongolia, China.	Xi’an and inner Mongolia China,	16S rRNA sequencing	Gastric mucosal samples	Five GC-enriched bacterial taxa whose species identifications correspond to Peptostreptococcus stomatis, Streptococcus anginosus, Parvimonas micra, Slackia exigua and Dialister pneumosintes had significant centralities in the GC ecological network	Coker et al., 2018
Hu	2018	6 patients with gastric cancer and 5 patients with superficial gastritis	Beijing, China	Shotgun metagenomic sequencing	Gastric wash samples	The most representative taxa found in gastric cancer are members of known commensal or opportunistic pathogenic bacteria that typically colonize the oral cavity，including species Streptococcus_mitis_oralis_pneumoniae.	Hu et al., 2018
Chen	2019	62 patients with gastric cancer undergoing subtotal gastrectomy	Shenyang, China	16S rRNA sequencing	Cancer tissue and adjacent noncancer tissue	The genera Streptococcus, Peptostreptococcus were enriched in cancerous tissues.	Chen et al., 2019
Liu	2019	276 patients with gastric cancer who underwent gastrectomy without preoperative chemotherapy	Zhejiang Province, China	16S rRNA gene sequencing	230 were normal, 247 were adjacent noncancer tissue, and 229 were tumor tissue	In the tumor microbial environment, the abundances of Helicobacter pylori and Prevotella significantly decreased, while the abundance of Streptococcus anginosus increased significantly.	Liu et al., 2019
Shao	2019	67 cases of esophageal carcinoma and 36 cases of cardia adenocarcinoma underwent surgical treatment	Henan Province, China	16S rRNA next generation sequencing.	Tumor and adjacent nontumor tissues	At the genus level, the relative abundances of Prevotella, Streptococcus and Veillonella were higher in cardia adenocarcinoma tumor tissue than in nontumor tissue.	Shao et al., 2019
Gunathilake	2020	268 patients with gastric cancer and 288 healthy controls	Korea	16S rRNA gene sequencing	Gastric mucosa tissues	Streptococcus_NCVM species was highly abundant in GC cases	Gunathilake et al., 2020
Dai	2021	37 patients with gastric cancer in Zhejiang Province, China ，Validation in 20 gastric cancer patients in Jiangxi Province，China.	Zhejiang Province and Jiangxi Province，China	16S rRNA gene sequencing	Cancerous tissue and gastric antrum mucosa at a distance of 5 cm from the cancerous tissue	Increased abundances of Lactobacillus, Streptococcus, and Prevotella genera in cancerous tissue.	Dai et al., 2021
Gunathilake	2021	268 cases of early gastric cancer and 288 healthy controls	Korea	16S rRNA gene sequencing	Mucosal tissue at 3 cm from the tumor, gastric antrum and gastric corpus mucosa in the control group	The abundances of Streptococcus vestibularis and Peptostreptococcus stomatis decreased significantly in gastric cancer group	Gunathilake et al., 2021
Pimentel-Nunes	2021	Patients with normal stomach (control group, 25), advanced atrophic gastritis with intestinal metaplasia (IM, 18) and early gastric cancer (EGC, 34)	Portugal	16S rRNA next generation sequencing.	Gastric antrum and corpus biopsy specimens	From control to IM, then to EGC, the abundances of two bacteria gradually increased: Gemella from 1.48 to 3.9%; Streptococcus from 19.3 to 33.7%, being the dominant bacteria in EGC. At the species level, even though several streptococcus increased from normal mucosa to cancer, Streptococcus anginosus, Streptococcus oralis and Streptococcus mitis were the more prevalent and frequent in cancer patients	Pimentel-Nunes et al., 2021

### Changes in intestinal Streptococcus during the occurrence and development of gastric cancer

Studies have found that the colonic environment is completely different from the oral and gastric environments in terms of biological and ecological characteristics (Tsuda et al., 2015), and microorganisms in the stomach of healthy people will affect the results of fecal microbiological analysis (Stearns et al., 2011). Several studies have shown that the intestinal flora changes during the occurrence and development of gastric cancer. Therefore, it is uncertain whether the gut, as a downstream organ of the stomach, is affected and causes changes in the intestinal flora, or whether changes in the intestinal flora promote the occurrence and development of gastric cancer. Fecal analysis is mainly used in the study of the intestinal flora because diet and lifestyle are key factors in the formation of gut microbes; thus, studies have shown that lifestyle has a great impact on gastric cancer risk and sex differences in gastric cancer (Zhang et al., 2013). Propensity score matching (PSM) can be used to eliminate the influence of lifestyle on data regarding the reliability and correlation of fecal bacteria and to increase the authenticity of research results. Studies using the INS-GAS mouse model have shown that the gut microbiota promotes the occurrence of gastric cancer (Bik et al., 2006; Maldonado-Contreras et al., 2011; Lertpiriyapong et al., 2014; Pinzon-Guzman et al., 2019), indicating that certain bacteria in the gut are associated with the occurrence of gastric cancer.

In recent years, researchers have paid attention to the role of gut microbes in the occurrence and development of gastric cancer. It is generally believed that intestinal Streptococcus is associated with the risk of gastric cancer and can be used as a potential marker for predicting gastric cancer (see Table 3). In 2019, a case–control study was conducted in Shanxi Province, China. Through the analysis of microorganisms in the feces of gastric cancer and healthy control groups, Streptococcus was found to be enriched in gastric cancer patients, and the AUC resulting from the use of Streptococcus to distinguish the gastric cancer from the healthy control group was 0.81, indicating that Streptococcus can be used as a potential marker for predicting gastric cancer (Qi et al., 2019). This is the first study to examine the relationship between intestinal Streptococcus and stomach cancer. The following year, a study in Jiangsu Province, China, found that some common oral community members (such as Streptococcus mitis and Streptococcus salivarius subsp) in stool specimens were associated with the risk of gastric cancer (Wu et al., 2020). Subsequently, researchers from other provinces in China also performed 16S rDNA sequencing and 16S rRNA sequencing on the stool of patients with gastric cancer and found that Streptococcus abundance was increased in the intestinal flora of patients with gastric cancer, and the difference was statistically significant (Zhang Y. et al., 2021; Liu S. et al., 2021; Zhang Z. et al., 2021). Moreover, a Japanese study found that the intestinal microflora after surgery for gastric cancer also changed, with Streptococcus becoming the dominant bacteria (Erawijantari et al., 2020), which was similar to the results of other studies on the changes in gastric microflora observed after surgery. Yu et al. further compared the changes in bacteria in the feces between patients with gastric cancer and healthy controls, as well as patients with liver metastasis and nonliver metastasis, and found that Streptococcus was enriched in the gastric cancer group; Streptococcus was also identified as a microorganism that could predict liver metastasis of gastric cancer by comparing the liver metastasis group (L group) with the nonhepatic metastasis group (M group). However, survival analysis suggested that Streptococcus was not a prognostic factor for gastric cancer (Yu et al., 2021).

**Table 3:** The study of intestinal Streptococcus in gastric cancer.

Year	Author	Subjects	Region/country	Method	Samples	Main findings	Reference
2019	Qi	116 patients with gastric cancer and 88 healthy controls	Shanxi Province, China	16S rRNA gene sequencing	Feces	12 bacterial genera, including Lactobacillus and Streptococcus, were enriched in GC.	Qi et al., 2019
2020	Wu	134 patients with gastric cancer and 58 matched healthy controls	Jiangsu Province, China	16S rRNA and 18S rRNA gene sequencing	Feces	Streptococcus mitis and Streptococcus salivarius subsp. in stool specimens were associated with the risk of gastric cancer.	Wu et al., 2020
2021	Liu	38 patients with gastric cancer and 35 healthy volunteers	Shandong Province, China	16S rRNA gene sequencing	Feces	The facultative anaerobic (aerotolerant) bacteria were Enterobacteriaceae, Escherichia and Streptococcaceae, and the abundances of all were elevated in the intestine of gastric cancer patients	Liu S. et al., 2021
2021	Yu	49 patients with gastric cancer (C group), 49 healthy control (N group)，26 patients were divided into liver metastasis group (L group) and nonliver metastasis group (M group)(n = 13).	Wuhan, China	16S rRNA gene sequencing	Feces	At the genus level, lactobacillus and streptococcus were enriched in group C. By comparing group L with group M, streptococcus was identified as a microorganism that could predict liver metastasis of gastric cancer.	Yu et al., 2021
2021	Zhang	83 cases of noncardia gastric cancer, 54 cases of chronic atrophic gastritis, 29 cases of colorectal cancer and 61 healthy individuals	Zhejiang Provence, China	16S rRNA gene sequencing	Feces	The abundance of the Streptococcus genus was increased in the intestinal microbiota of gastric cancer patients.	Zhang Y. et al., 2021
2021	Zhang	22 patients with gastric cancer and 30 healthy (Hp negative, no gastrointestinal symptoms)	Qinghai Province, China	16S rDNA gene sequencing	Feces	At the genus level, Prevotella, Streptococcus and Lactobacillus abundances were higher in the gastric cancer group than in the healthy group and the difference was statistically significant.	Zhang Z. et al., 2021

## Possible carcinogenic mechanism of Streptococcus

Studies have shown that different species of Streptococcus play an important role in cancer, affecting the occurrence and development of tumors through various metabolite changes and regulation of the immune microenvironment (Morita et al., 2003; Narikiyo et al., 2004; Abdulamir et al., 2011; Moritani et al., 2015; Zhou et al., 2017; Sheikh et al., 2020). Streptococcus is enriched in gastric cancer and is the dominant bacteria in gastric cancer flora. Many studies have studied gastric cancer flora as a whole and found that it is associated with changes in various metabolic pathways and the immune microenvironment. Studies have found that purine metabolic pathways are enriched in gastric cancer, suggesting that the gastric cancer microbiome metabolizes and releases purines in the tumor microenvironment (Coker et al., 2018; Chen et al., 2019) and that purines regulate the immune cell response and cytokine release (Di Virgilio, 2012). The LPS (lipopolysaccharide) biosynthetic pathway is enriched in gastric cancer (Hu et al., 2018), and LPS can promote an inflammatory response in the tumor microenvironment (Rakoff-Nahoum and Medzhitov, 2009; Gagliani et al., 2014), suggesting that the gastric microbiota promotes inflammation. The activation of some pathways that contribute to cell recognition is reduced in gastric cancer, such as bacterial motility and signal transduction pathways (Coker et al., 2018; Chen et al., 2019). In a comparative analysis of gastric cancer and chronic gastritis patients in Portugal and Mexico, Ferreira et al. found that the activities of nitrate reductase and nitrite reductase in gastric cancer flora increased (Ferreira et al., 2018), thereby increasing levels of nitrite, which is the precursor of carcinogen NOC (Correa, 1992). There are also studies showing that the activation of some amino acid metabolic pathways, such as those for isoleucine and valine, is increased in gastric cancer (Jung et al., 2014; Wang H. et al., 2016; Hu et al., 2018; Liu et al., 2019; Gunathilake et al., 2020; Huang et al., 2021). Hp is present in the gastric cancer flora, as well as other bacteria, so the changes in metabolic pathways are not necessarily caused by Streptococcus.

**Figure 3:**
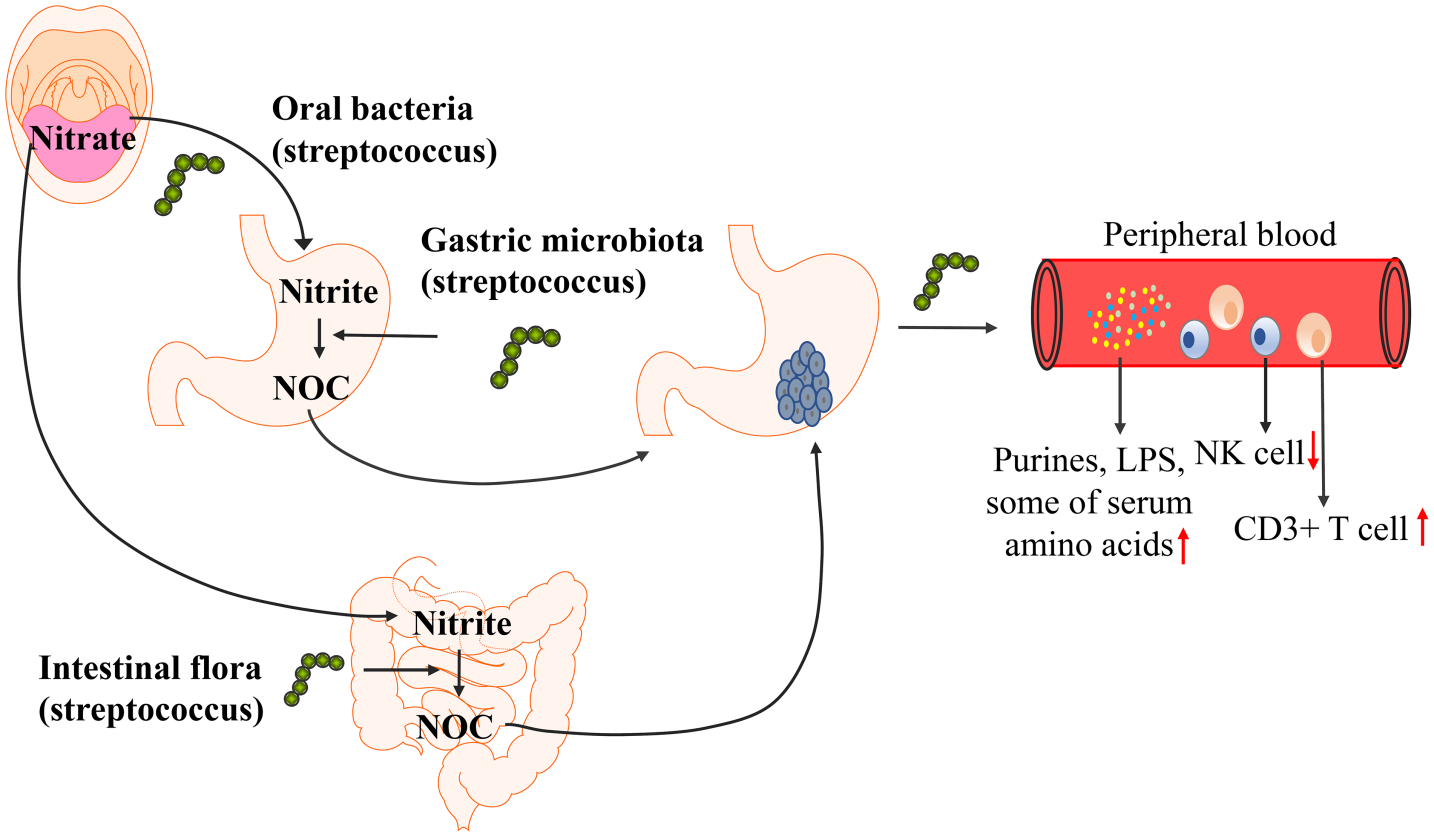
Possible carcinogenic mechanism of Streptococcus. The LPS (lipopolysaccharide) biosynthetic pathway and purine metabolic pathways are enriched in gastric cancer. Streptococcus abundance was positively correlated with the levels of serum amino acids (L-alanine, L-threonine, methionine, L-carnitine, guanidinoacetate), glutathione, cysteine, and methionine levels. Streptococcus was positively correlated with the number of CD3+ T cells and negatively correlated with the number of NK cells. Streptococcus is involved in the formation of NOC (nitroso compounds) in the stomach, and the formation of NOC increases the risk of gastric cancer.

To further determine the carcinogenic mechanism of Streptococcus, researchers separately analyzed the correlation between changes in Streptococcus abundance and metabolites and the regulation of the immune microenvironment to determine the carcinogenic mechanism of Streptococcus. Studies have found that Streptococcus is involved in the formation of NOC (nitroso compounds) in the stomach (Ayanaba and Alexander, 1973; Jo et al., 2016; Sohn et al., 2017), and the formation of NOC increases the risk of gastric cancer (Ayanaba and Alexander, 1973; Mowat et al., 2000; Dicksved et al., 2009; Jo et al., 2016). Streptococcus is associated with a variety of metabolic changes. Wu et al. found that Streptococcus abundance was positively correlated with the levels of serum amino acids (L-alanine, L-threonine, methionine, L-carnitine, guanidinoacetate), heptanal and phenylethylamine by analyzing serum metabolites (Wu et al., 2020). Dai et al. found that Streptococcus abundance was positively associated with glutathione, cysteine, and methionine levels, and the activation of these metabolic pathways was increased in gastric cancer (Dai et al., 2021). In addition to studying metabolites, streptococcal infection may also affect the immune microenvironment in the body. Qi et al. studied the changes in immune cells in peripheral blood and found that the abundance of Streptococcus was positively correlated with the number of CD3+ T cells and negatively correlated with the number of NK cells (Qi et al., 2019). The possible carcinogenic mechanism of Streptococcus is shown in Figure 3. The correlation between Streptococcus and metabolic pathways and the immune microenvironment has only been studied in recent years, and few research results have been achieved; the specific mechanism has not been further explored. Therefore, how Streptococcus affects the occurrence and development of gastric cancer is still unclear.

## Summary and prospects

In summary, streptococci are common bacteria in the digestive tract and were first discovered in the stomach. However, due to the immature technology and incomplete understanding at that time, people paid more attention to the pathogenicity of Hp in gastric cancer. With the development of microbial detection technology and progress in scientific knowledge, Streptococcus has been discovered and studied in depth by many researchers. Streptococcus was found to be affected by Hp and gastric acid in the stomach of nongastric cancer patients, while in the absence of Hp, Streptococcus abundance in gastric cancer patients was prominent, indicating that Streptococcus may act together with Hp or play a role in different stages of gastric cancer. Streptococcus changes in different parts of the digestive tract in patients with gastric cancer. We describe the changes in Streptococcus in the oral cavity, stomach and intestinal tract. In the oral cavity, at the genus level, Streptococcus was enriched in gastric cancer patients; at the species level, some species of Streptococcus exhibited reduced abundances. In the stomach, at the genus level, the abundance of Streptococcus was significantly increased in gastric cancer patients; at the species level, the changes in different species of Streptococcus were different. In the gut, at the genus level, Streptococcus abundance in gastric cancer patients increased significantly, which was the same as that in the oral cavity and stomach, while Streptococcus abundance varied at the species level. Streptococcus can affect various metabolic pathways and the immune microenvironment of gastric cancer and play an important role in its occurrence and development. However, the causal relationship between Streptococcus and gastric cancer has not been established, nor has the pathogenesis been determined.

Early-stage gastric cancer has a good prognosis, but most patients already have advanced gastric cancer when they are first diagnosed (Smyth et al., 2020), and the 5-year survival rate is less than 30% (Huang et al., 2021). Gastric cancer is mainly diagnosed by gastroscopy and pathological biopsy. Due to the invasiveness of gastroscopy, it cannot be popularized as an early diagnosis method in the population, and other detection methods that can be widely used and that effectively predict early-stage gastric cancer are needed. The stomach, as an important organ of the digestive tract, has been studied to assess the carcinogenic mechanism of Hp in the early years due to its special environment and the presence of Hp colonization, but the pathogenicity of other microorganisms in gastric cancer has been ignored. Due to the development of molecular biology and gene detection technology, microbial research is no longer limited to traditional bacterial culture, and due to the application of metagenomics to microbial research, scientists have a more systematic and comprehensive understanding of microorganisms, so the study of the relationship between Streptococcus and tumors has become a hot spot in recent years. Streptococcus has been found to be carcinogenic in esophageal cancer and colorectal cancer (Abdulamir et al., 2011; Wang et al., 2012; Tsai et al., 2016; Liu et al., 2018; Guven et al., 2019; Kawasaki et al., 2021).

The study of Streptococcus in patients with gastric cancer is not as advanced as that of other gastrointestinal tumors, but great progress has been made in recent decades. Streptococcus overgrowth in the oral cavity, stomach and intestine of gastric cancer patients affects metabolites and peripheral immune cells and is a potential biomarker that can be used to assist in the diagnosis of gastric cancer. It is also a possible therapeutic target, providing new ideas for the treatment of gastric cancer. Intragastric Streptococcus affects the gastric microenvironment, but its pathogenic mechanism in gastric cancer remains unclear. Streptococcus in the oral cavity and intestine may be a potential predictor of gastric cancer. The characteristics of easy collection, low cost and noninvasiveness during the acquisition of specimens indicate that the assessment of Streptococcus may become a screening method for early-stage cancer. The current dilemma facing the use of Streptococcus as a treatment target has three components: (1) the pathogenicity and pathogenesis of Streptococcus have not been determined; (2) whether the use of traditional antibiotic treatment will destroy the microecological environment and cause other adverse events due to dysbiosis has not been determined; and (3) the use of probiotics may become a treatment method, but there is no relevant research thus far. Future research can be carried out from two aspects: (1) using animal experiments, an INS-GAS mouse model can be used to clarify the role of Streptococcus in gastric cancer; (2) using clinical studies, including descriptive and cross-sectional studies and function-based studies and prospective studies, studying the effects of Streptococcus and its metabolites in the digestive tract will be helpful for an in-depth understanding of its pathogenesis. However, since the development of gastric cancer takes decades and less than 3% of the H. pylori-infected population eventually develops gastric cancer, longitudinal studies and prospective studies are difficult to achieve. Microbial research on gastric cancer still has far to go, but assessments of Streptococcus, as a noninvasive auxiliary diagnostic method, will usher in a qualitative leap with the efforts of many scientists. If it is successfully applied to the clinic, it will greatly improve the early diagnosis rate and change the future of gastric cancer.

## Author contributions

XDC and LY conceptualized the manuscript. MLZ, YQZ, CH, SJZ, and JXC collected the literature, MLZ and YQZ collected the literature, wrote the manuscript and made the figures. XDC and LY edited and made significant revisions to the manuscript. All authors contributed to the article and approved the submitted version.

## Funding

This study was supported by Natural Science Foundation of Zhejiang Province (HDMY22H160008), Medical Science and Technology Project of Zhejiang Province (2022KY114 and WKJ-ZJ-2104), Chinese Postdoctoral Science Foundation (2022M713203), Program of Zhejiang Provincial TCM Sci-tech Plan (2022ZQ020), Science and Technology Projects of Zhejiang Province (2019C03049), National Natural Science Foundation of China (82074245 and 81973634), and Zhejiang Provincial Research Center for Upper Gastrointestinal Tract Cancer (JBZX-202006).

## Conflict of interest

The authors declare that the research was conducted in the absence of any commercial or financial relationships that could be construed as a potential conflict of interest.

## Publisher’s note

All claims expressed in this article are solely those of the authors and do not necessarily represent those of their affiliated organizations, or those of the publisher, the editors and the reviewers. Any product that may be evaluated in this article, or claim that may be made by its manufacturer, is not guaranteed or endorsed by the publisher.
